# Generative artificial intelligence in medicine: a mixed-methods survey of UK general practitioners

**DOI:** 10.1136/bmjdhai-2025-000051

**Published:** 2025-07-23

**Authors:** Anna Kharko, Cosima Locher, John Torous, Sophie Anna Rosch, Maria Hägglund, Jens Gaab, Brian McMillan, David Sundemo, Kenneth D Mandl, Charlotte Blease

**Affiliations:** 1 Department of Women's and Children’s Health, Uppsala University, Uppsala, Sweden; 2 Centre for Primary Care and Health Services Research, The University of Manchester, Manchester, UK; 3 Department of Consultation-Liaison Psychiatry and Psychosomatic Medicine, University of Zurich, Zürich, Switzerland; 4 Department of Clinical Psychology and Psychosomatics, University of Basel, Basel, Switzerland; 5 Department of Psychiatry, Harvard Medical School, Boston, Massachusetts, USA; 6 University Hospital Zurich, Zürich, Switzerland; 7 Clinical Psychology and Psychotherapy, University of Basel, Basel, Switzerland; 8 Institute of Medicine, University of Gothenburg, Gothenburg, Sweden; 9 Boston Children’s Hospital, Harvard Medical School, Boston, Massachusetts, USA

**Keywords:** Artificial intelligence, Documentation, Primary Health Care

## Abstract

**Objective:**

To explore the opinions of general practitioners (GPs) in the UK about the use of generative artificial intelligence (AI) tools in primary care.

**Methods and analysis:**

At the beginning of 2024, using a convenience sample, we administered an online mixed-methods survey to registered GPs currently working in the UK.

**Results:**

A total of 1006 GPs responded, with 53% being male and 54% over 46 years old. One-fifth of GPs reported having used AI for clinical practice, with male doctors and those in bigger cities being more likely to have used it. 80% of respondents expressed a need for more training in understanding these tools. GPs at least somewhat agreed AI would improve documentation (59%) and patient information gathering (56%). 55% felt AI could increase inequities and 54% saw potential for patient harm, but 47% believed it could enhance healthcare efficiency. GPs who used these tools were significantly more optimistic about the scope for generative AI in improving clinical tasks. One-third of GPs left comments that were classified into four major themes: (1) lack of familiarity and understanding with AI, (2) role of AI in clinical practice, (3) concerns about AI and (4) AI and the future of healthcare.

**Conclusions:**

This study highlights UK GPs’ developing perspectives on generative AI in clinical practice, emphasising the need for more training. Many GPs reported a lack of knowledge and experience with this technology, although a portion already used non-medical grade technology for clinical tasks, with the risks that this entails.

**WHAT IS ALREADY KNOWN ON THIS TOPIC**

- Artificial intelligence (AI)-powered chatbots, like ChatGPT, are gaining interest in medicine, particularly for clinical documentation and diagnostic support.
- Existing research highlights potential benefits of AI tools in reducing administrative burdens, yet regulatory, ethical and privacy challenges remain.

**WHAT THIS STUDY ADDS**

- Exploring general practitioners’ (GPs’) opinions, we found they anticipated a variety of benefits from AI, including aiding in documentation, patient information gathering and diagnosis.
- Concerns among GPs were also prominent, including legal risks, patient harm and increasing healthcare inequities as a result of using AI.
- 80% of GPs expressed the need for more training in AI applications.

**HOW THIS STUDY MIGHT AFFECT RESEARCH, PRACTICE OR POLICY**

- Against GPs’ optimism, and their concerns, and given that some GPs are already using AI in clinical practice, the urgency for clear guidelines, robust training and effective regulation is paramount.

## Introduction

A growing body of research shows that documentation and administrative tasks are a leading source of burnout among all clinicians including primary care physicians, many of whom anticipate generative artificial intelligence (AI) will soon provide assistance.^1 2^ Tools based on generative AI, like large language model (LLM)-powered chatbots, may be especially well-equipped to help with these tasks.^3 4^ These tools have particular strengths in summarising outputs, and they can also do so in a requested style, literacy level or socioemotional tone, suggesting scope for assistance with clinical documentation, including notes that patients may read.^5 6^ Already, some versions of AI such as Nuance’s Dragon Ambient eXperience can ‘listen’ into the appointment and translate the dialogue between doctor and patient into clinical notes.^7^ So far, it is unclear whether these tools improve practice efficiencies, but preliminary findings are promising.^8 9^ A growing body of research indicates that LLM tools could have considerable scope in assisting with patient history-taking.^10^

LLM chatbots, however, have limitations and invite novel concerns. They are unable to discriminate the quality of the information on which they are trained, and outputs can be plain wrong, and more worryingly, subtly inaccurate.^11^ Such incorrect information, conveyed in an authoritative tone, can potentially lead to patient harm.^8 12^ These models may also risk ‘algorithmic discrimination’ via the creation of unfair biases in their recommendations.^13 14^ It is unclear whether these risks are worse than the ones that already exist with human-mediated care.^15^ Findings indicate that these models could compound inequity problems^16^; however, other research suggests that there is promising scope for this AI to be employed, constructively, as a tool to reduce clinical inequities.^17 18^

Beyond output accuracy, patient privacy can also be put at risk with the adoption of some LLM-powered chatbots in clinical contexts.^19^ Owing to their ease of use, conversational fluency and perhaps the tendency of users to anthropomorphise these tools, patients and clinicians may be tempted to input sensitive, potentially personally identifiable clinical information.^20^ While medical-grade chatbots that comply with healthcare privacy standards exist, with many more under development,^21^ readily available commercial chatbots such as OpenAI’s ChatGPT that are not designed for clinical purposes increasingly attract more users. Some medical organisations have issued position statements cautioning that physicians should not be using these tools to undertake clinical work.^22 23^ Still, preliminary evidence suggests some physicians may be adopting LLM-powered chatbots for a variety of tasks including assisting with documentation.^24 25^ A study of 420 US medical trainees found that 40% had used ChatGPT.^26^ In an earlier report emerging from the GPAI-UK-2024 Survey, we found that one-fifth of general practitioners (GPs) in the UK (20%; 205/1006) had used generative AI to assist with some aspect of clinical practice.^27^ It is likely that the trend for increased uptake continues as doctors seek out solutions to alleviate administrative burdens. Yet, there is a dearth of research into the perspectives of practising clinicians on these tools.

The present publication builds on the short report of the GPAI-UK-2024 Survey.^27^ Our aim was to explore GP’s opinion, expectations and concerns about generative AI in primary care, as well as to analyse what GP individual characteristics, if any, are associated with this early adoption of generative AI tools and opinions about them.

## Material and methods

The study is reported in accordance with the Checklist for Reporting Results of Internet E-Surveys (CHERRIES) checklist (see online supplemental appendix 1).

10.1136/bmjdhai-2025-000051.supp1 Supplementary data

### Study design and setting

We conducted an anonymous nationwide, mixed-methods, web-based survey of GPs in the UK. The study employed a convenience sample to solicit the opinions of GPs using the clinician marketing service Doctors.net.uk.^28^ This is the largest professional network and online information service for doctors in the UK; it is free to join and requires doctors to give their General Medical Council (GMC) registration details.^28^ Currently, Doctors.net.uk has 254 741 members out of a total of approximately 379 208 registered doctors in the UK (67%).^29^ The study was selected to be part of an ‘omnibus’ monthly survey administered by Doctors.net.uk which focuses on topical medical issues and has predetermined sample sizes of 1000 participants as this is in line with other sample sizes of healthcare providers.^30^ The study team has obtained samples from Doctors.net.uk in previous studies using similar methods.^1 2 31^

At Doctors.net.uk, a percentage of GPs active within the community consent to being sent survey invitations via email, which differs according to those who are active on the website at any given period. Depending on how GPs consented to receive survey invitations, between 2 February 2024 and 22nd February 2024, our study was advertised via email and displayed on the Doctors.net.uk home pages or only displayed on doctors’ homepages, among a sample of GPs. During the survey administration period, approximately 21 000 GPs were active in the community.

We asked Doctors.net.uk to invite a random sample of GPs from across the UK. This sampling was stratified by regional location using demographic information about currently registered GPs working in the UK provided by the GMC in the GMC Data Explorer.^29^ Using this stratification, Doctors.net.uk embedded invitations within the homepages of 13 967 GPs who were active on the website in the last 90 days; of these, 2775 who consented to research communications were also sent an email to participate.

### Survey

The study team developed a survey instrument to examine GPs’ use and opinions about generative AI in clinical practice (see online supplemental appendix 2). The survey, which encompassed 24 items, was timed to take around 5 min to complete and pretested and piloted with 6 UK GPs to assess face validity. The survey was divided into three main sections and specifically asked about GPs’ experiences and opinions with respect to Open AI’s ChatGPT, Google’s Bard or Microsoft’s Bing AI. We emphasise that these AI tools are not specifically trained on medical data and recognise that there is currently a plethora of LLMs, including but not limited to Anthropic’s Claude-series, Meta’s Llama-models and Mistral’s AI-models. However, given the commercial availability of Open AI’s ChatGPT, Google’s Bard or Microsoft’s Bing AI at the time of data collection, we focused on them in the study.

10.1136/bmjdhai-2025-000051.supp2 Supplementary data

Part A of the survey requested demographic information, including participant gender, age, their GP role, the regional location of their practice and the number of patients in their general practice. Further screening questions were embedded within the survey to determine whether respondents were currently registered and practising as GPs in the UK. Part B of the survey examined GPs’ experiences with using generative AI to assist with any aspect of clinical practice and, as noted, responses to this section are published separately as a brief report.^27^ Finally, part C requested participants to offer their opinions about these generative AI tools in the delivery of care. This section used a mixture of closed-ended Likert scale survey items and included two optional open-ended questions. All participants were requested to answer all the closed-ended questions and participants were advised they could leave the survey at any time.

### Patient and public involvement

No patients or public were involved in the study.

### Quantitative analysis

After survey collection, quantitative data were analysed using JASP (V.0.9.2; University of Amsterdam), RStudio (V.2024.04.2+764) and SPSS (V.27; IBM). We summarised respondents’ characteristics and opinions on Likert survey items through descriptive statistics (count, percentage). We also compared our sample of GPs to those on the GMC registry based on age, gender and practice location. To explore the impact of previous experience with AI on opinions about generative AI, we divided respondents into those who have used AI to assist in clinical practice and those who have not (item Q1, see online supplemental appendix 2). We analysed the impact of AI use on AI potential for improvement (item Q2) as well as impact on patients and clinicians (item Q3) using χ^2^ test of independence. To analyse if opinions on these items were associated with a respondent’s gender (‘women’ or ‘men’) or age (‘younger GPs’ defined as ≤45 years and ‘older GPs’ defined as ≥46 years), we carried out a Mann-Whitney U test. As part of this test, we calculated median ratings and excluded ‘don’t know’ responses due to data being ordinal opinion ratings. Data were checked for low-effort responses by calculating per-participant SD for items Q2 and Q3. No individual participant was flagged, and no data were thus removed. Lastly, we analysed what GP characteristics affected use of generative AI. We conducted a bidirectional stepwise regression analysis to investigate the predictors of AI usage in clinical practice. The outcome variable was item Q1 on whether respondents used AI tools in their clinical practice (0=not used AI, 1=used AI). Predictor variables included gender (male; female; other; prefer not to say), age (35 or under; 36–45; 46–55; 56 or over), practice size (up to 5000 patients; 5001–7500 patients; 7501–10 000 patients; 10 001–12 500 patients; 12 501 patients or more), role (GP partner/principal; salaried GP; locum GP; GP registrar; secondary care doctor; other), and practice location (major conurbation; large town/city; medium town/city; small town/city; village/hamlet; other). For this analysis, participants’ data were excluded if they did not indicate their gender (prefer not to say; n=8). Predictors were added or removed stepwise based on Akaike’s information criterion (AIC). The full model included interactions between gender and the other predictors, while the null model included no predictors. The most parsimonious model with the lowest AIC value was selected.

### Qualitative analysis

This study followed a concurrent triangulation mixed-methods design, with a predominant quantitative component, while qualitative data were obtained from the two optional open-ended questions. These allowed participants to respond in detail to the topic of the questionnaire: ‘*Please add any additional comments you might have about the use of Chat GPT/Bard/Bing AI in primary care’* (Item Q5, see online supplemental appendix 2), and ‘*Please add any additional comments you might have about the use of the Chat GPT/Bard/Bing AI in medicine’* (Item D3). We carried out inductive thematic coding of the fully anonymised data.^32^ Responses were analysed by two members of the research team (CB and JT). CB is a philosopher of medicine, ethicist and health informatician from the UK, and JT is a psychiatrist and health informatician from the USA. The transcripts were initially read several times to achieve familiarisation with participants’ responses. Next, an inductive coding process was used, in which brief descriptive labels (‘codes’) were applied to each comment. Multiple codes were applied to the comments with multiple meanings. Comments and codes were reviewed and compared with investigate similarities and differences. As a result of this iterative process, first-order codes were grouped into second-order themes. CB and JT met to discuss coding decisions, and subsequently, minor revisions were made.

## Results

### Quantitative analysis

Of the 2775 who received the email, 2683 opened the email invite (97%) and 604 (22%) clicked on the survey link with 532 (19%) completing the survey (see online supplemental appendix 3). The other half of the final sample (n=474) accessed and completed the survey via their homepage (474/11 192, 4%). Of the total number of 1006 GPs who responded, 53% were male, and 54% were aged 46 years or older (see table 1).

10.1136/bmjdhai-2025-000051.supp3 Supplementary data

**Table 1:** Respondents’ characteristics

Characteristic	Total (N=1006)
Gender, n (%)	
Female	467 (46.42)
Male	531 (52.78)
Prefer not to say	8 (0.8)
Age, n (%)	
35 or under	76 (7.55)
36–45	386 (38.37)
46–55	348 (34.59)
56 or over	196 (19.48)
Practice location, n (%)	
Large town/city (eg, Nottingham, Cardiff)	182 (18.09)
Major conurbation (eg, London, Glasgow)	172 (17.10)
Medium town/city (eg, Worcester, Dundee)	224 (22.27)
Small town/city (eg, Thetford, Omagh)	305 (30.32)
Village/hamlet	115 (11.43)
Out of hours area	3 (0.3)
Rural/remote	1 (0.1)
Other*	4 (0.4)
Practice size, n (%)	
Up to 5000 patients	117 (11.63)
5001–7500 patients	149 (14.81)
7501–10 000 patients	213 (21.17)
10 001–12 500 patients	183 (18.19)
12 501 patients or more	344 (34.19)

*For practice location, free-text answers to option ‘other’ included: HMPS prison (n=1), health board (n=1), military garrison (n=1) and national (n=1).

HMPS, His Majesty's Prison Service.

Requested to describe their role, almost half reported ‘GP partner/principal’ (45%; 455/1006), followed by ‘salaried GP’ (34%; 341/1006), ‘locum GP’ (18%; 181/1006), and lastly ‘GP registrar’ (3%; 29/1006). As seen in table 1, a third were based in a ‘small town/city’ (30%) and a third had ‘12 501 or more’ patients listed in the practice (34%).

Our participant pool exhibited some distinctions from the GPs registered with the GMC in the UK. We had an almost equal gender split while the majority of GPs in the GMC registry were female, with further differences observed for age and regional distributions (see online supplemental appendix 3).

#### GPs’ opinions about the impact of generative AI

**Figure 1:**
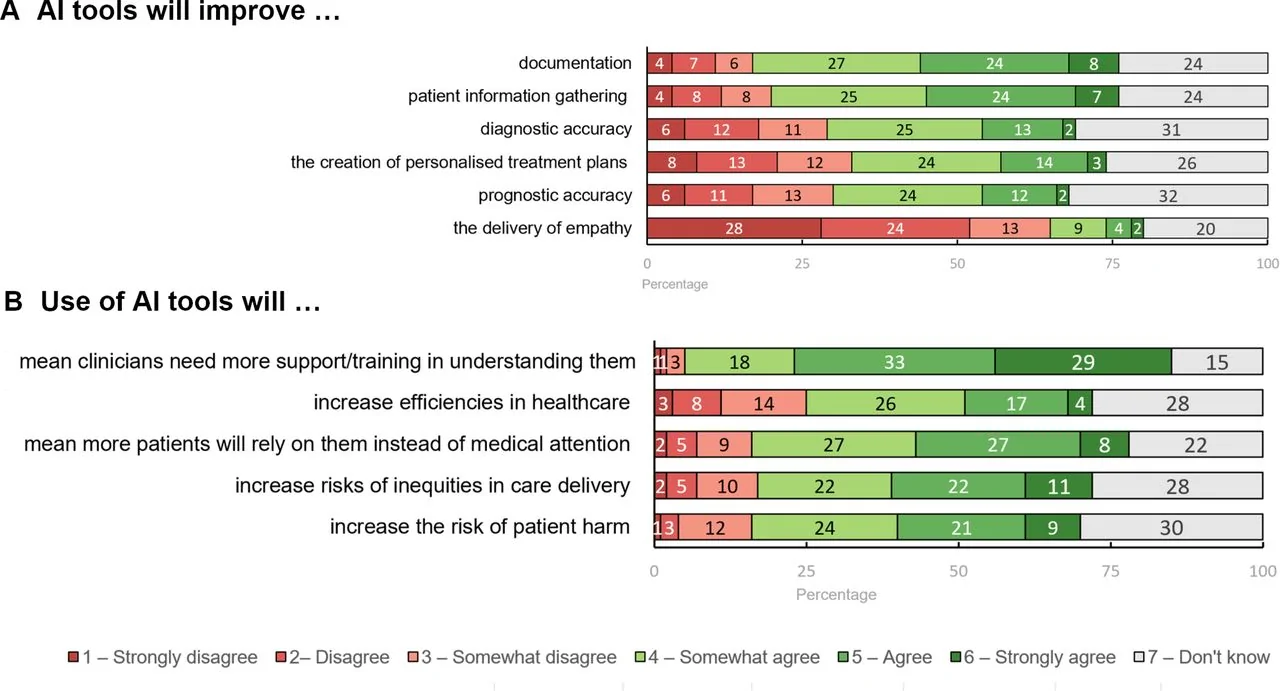
(A) Distribution of responses to the potential for ChatGPT/Bard/Bing AI to improve clinical tasks, across the whole sample. (B) Distribution of responses to the potential impact of ChatGPT/Bard/Bing AI on care delivery, across the whole sample. AI, artificial intelligence.

GPs reported a variety of opinions about the impact of generative AI on clinical tasks with at least one in five respondents reporting ‘Don’t know’ (see figure 1A). Overall, however, GPs were more positive than negative about the potential impact of AI across all tasks except for empathy, where two-thirds disagreed.

When considering how the use of AI will impact patients and clinicians, overwhelmingly GPs agree that clinicians will need additional support and training (see figure 1B). Although GPs continued to be uncertain on the effects of AI, as evident in the relatively large portion of ‘don’t know’ answers, they agreed on more potential negatives. For example, half (55%) expected increased risks of inequalities and half (54%) foresaw increased risk of patient harm as a result of using AI tools.

When asked for their opinions on possible litigation risks associated with the use of generative AI, across the whole sample, 2% (n=22) reported these tools would ‘decrease my risk of having legal action taken against me’, 34% (n=340) believed that use would ‘neither decrease nor increase my risk’ and 20% (n=201) believed that use would ‘increase my risk of having legal action taken against me’. Almost half said that they ‘didn’t know’ 44% (44%, n=443).

#### GP characteristics associated with use of generative AI

205 (20%) GPs reported to have used AI for clinical purposes. There were differences in participant characteristics between those who used AI and those who did not (see table 2).

**Table 2:** Differences between GPs’ characteristics based on use of AI

	Used AI (n=205)	Not used AI (n=801)
Gender, n (%)		
Female	73 (35.6)	394 (49.2)
Male	129 (62.9)	402 (50.2)
Prefer not to say	3 (1.5)	5 (0.6)
Age, n (%)		
35 or under	20 (9.8)	56 (7)
36–45	76 (37.1)	310 (38.7)
46–55	68 (33.2)	280 (35.0)
56 or over	41 (20.0)	155 (19.4)
Practice location, n (%)		
Major conurbation	43 (21.0)	129 (16.1)
Large town/city	45 (22.0)	136 (17.0)
Medium town/city	56 (27.3)	168 (21.0)
Small town/city	50 (24.4)	255 (31.8)
Village/hamlet	11 (5.3)	104 (13.0)
Other	0 (0)	9 (1.1)
Practice size, n (%)		
Up to 5000 patients	21 (10.2)	96 (12.0)
5001–7500 patients	28 (13.7)	121 (15.1)
7501–10 000 patients	45 (22.0)	168 (21)
10 001–12 500 patients	37 (18.0)	146 (18.2)
12 501 patients or more	74 (36.1)	270 (33.7)

AI, artificial intelligence; GPs, general practitioners.

Analysing the characteristics correlated with GPs reported use of generative AI in clinical practice, we carried out a regression. Based on the AIC statistic, the model that best explained the variance in AI use consisted of gender and practice location, with female and medium town/city as reference categories (see online supplemental appendix 3, equation 1), F=5.499, df=6991, adj. R^2^=0.26, p<0.001. Male GPs were significantly more likely than female GPs to have used AI, as well as those practising in bigger cities (see online supplemental appendix 3).

#### GPs’ opinions based on their reported use of generative AI

**Figure 2:**
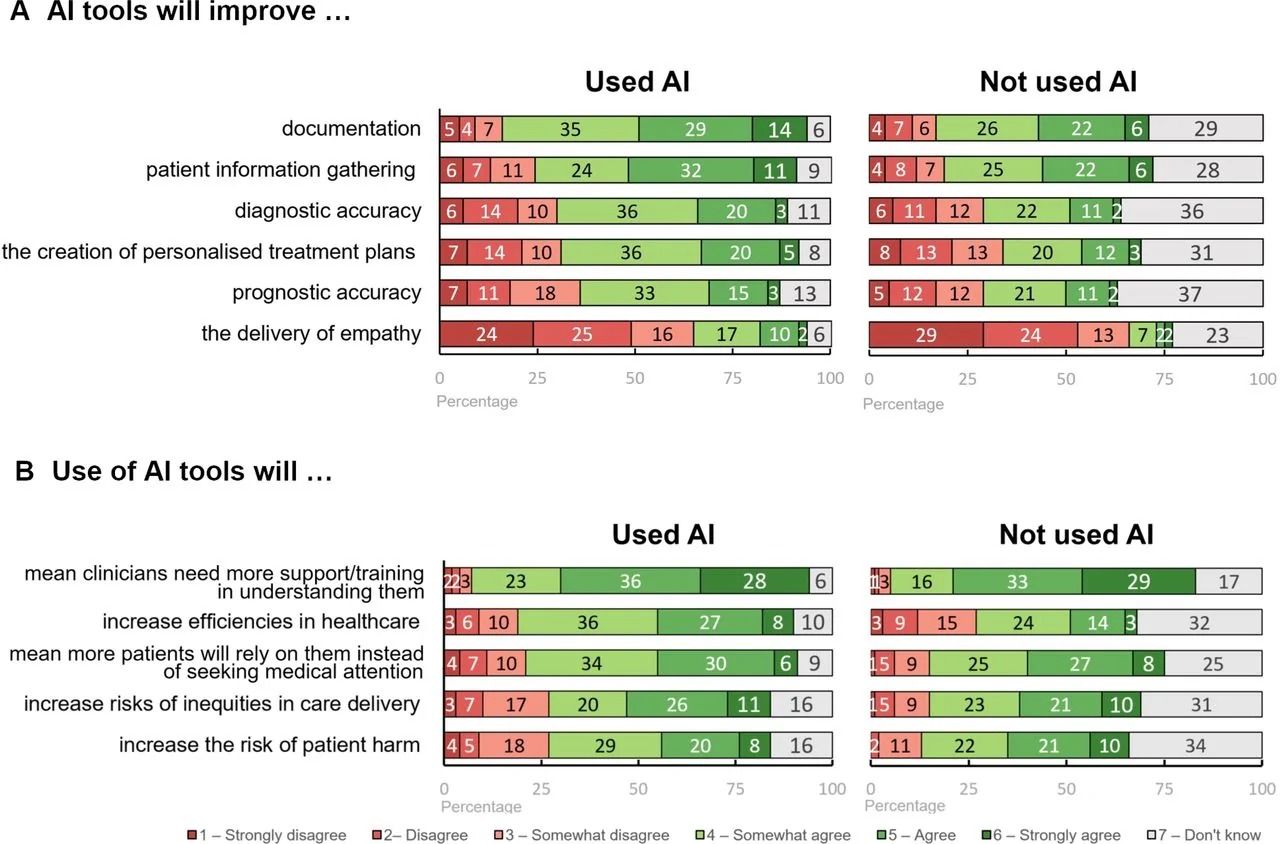
(A) Distribution of agreement levels to the survey item ‘AI tools will improve’ between GPs who have used generative AI to assist in clinical practice and GPs who have not. (B) Distribution of agreement levels to the survey item ‘Use of AI tools will’ between GPs who have used generative AI to assist in clinical practice and GPs who have not. AI, artificial intelligence; GPs, general practitioners.

When comparing between respondents who reported using or not using AI for assistance in clinical practice, significant differences emerged for all survey items describing AI’s potential for improvement with clinical tasks (see online supplemental appendix 3). GPs who had previously used generative AI for clinical purposes tended to anticipate greater potential of AI to improve clinical tasks than GPs who did not report using it, with the latter also providing more ‘don’t know’ answers (see figure 2).

Significant differences also emerged for all survey items describing generative AI’s potential to impact patients and clinicians (see online supplemental appendix 3). GPs who reported using generative AI in clinical practice tended to anticipate both more positive and fewer harmful effects and provided fewer ‘don’t know’ answers than those who did not report using it (see figure 2).

Lastly, GPs’ opinions about risk of litigation also differed based on previous AI experience (see table 3). Most (35%) of those who used AI believed such tools would ‘neither decrease nor increase my risk’. Among GPs with no prior experience, half answered they did not know (48%) and a third expected it to increase. χ^2^ of independence indicated that these differences in answer distributions were significant (see online supplemental appendix 3).

**Table 3:** Expectations of litigation between GPs based on previous use of AI

Item	Used AI (n=205)	Not used AI (n=801)
These tools will …		
decrease my risk of having legal action taken against me.	13 (6.3%)	9 (1.1%)
neither decrease nor increase my risk.	72 (35.1%)	129 (16.1%)
increase my risk of having legal action taken against me.	64 (31.2%)	276 (34.5%)
Don’t know.	56 (27.3%)	387 (48.3%)

AI, artificial intelligence; GPs, general practitioners.

#### GPs’ opinions based on respondents’ characteristics

GPs’ opinions on generative AI were not associated with GPs’ gender or age on most opinion items (see online supplemental appendix 3). Significantly more male GPs agreed that generative AI tools will improve patient information gathering (U=62 926, p=0.01). More female GPs, in comparison, believed that such tools will increase risks of inequities in care delivery (U=73 174, p<0.001) and patient harm (U=66 947, p=0.015). Older GPs, aged 46 year and over, agreed significantly more that AI tools will improve patient information gathering (U=78 704, p=0.008), the creation of personalised treatment plans (U=76 768.5, p=0.003) and empathy delivery (U=86 711, p=0.039).

### Qualitative analysis

A total of 307 (31% out of the 1006 respondents) left comments in response to at least 1 question (6468 words in total). Comments were brief (typically, sentence fragments or 1–3 sentences). GP respondents who submitted comments to each question were not significantly different in terms of sex and age from those who did not submit comments; however, they did differ depending on whether they reported using generative AI in clinical practice, or the number of reported patients in their practice, with GPs who used AI leaving more comments.

Owing to the iterative thematic analysis process, four major categories of GPs’ views were identified in relation to generative AI in medicine: (1) lack of familiarity and understanding, (2) AI role in clinical practice, (3) concerns and (4) the future. These categories were further subdivided into 12 themes, which are described in subsequent sections with illustrative comments. Numbers in parentheses are identifiers that ascribe comments to individual participants; in addition, gender is marked as (male or female), GP role is marked as (‘partner/principal’, ‘salaried,’ ‘locum’ or ‘registrar), and whether GPs reported using any generative AI to assist in any aspect of clinical practice (Y or N).

#### Theme 1: lack of familiarity and understanding

##### No knowledge

A dominant category was lack of knowledge about generative AI tools. Multiple short comments stated, ‘not aware of these’, ‘no idea’ or ‘no knowledge’. Similarly, many GPs responded that they did not know enough to comment; for example, ‘I don’t know anything about how it works so have been unable to offer an opinion’ (#20, Female, Partner/Principal, N). Some were openly candid about their lack of knowledge in relation to general practice: ‘I honestly have never used any AI software and therefore find it hard to have insight’ (#228, Male, Locum, N), ‘I just don’t know how this will impact us’ (#995, Male, Partner/Principal, N). Some expressed anxieties but were open about their lack of awareness: for example, ‘I am fearful, but ignorant!’ (#529, Female, Partner/Principal, N). Relatedly, some GPs also identified a lack of training, or need for more education on generative AI, for example, ‘Need more training and information about them’ (#94, Female, Salaried, N), ‘No idea what it’s about and we haven’t been given any help/support/info regarding this’ (#45, Female, Salaried, N), and ‘I would love to learn more about this’ (#298, Female, Locum, N).

##### No experience

Another related category was a lack of experience with generative AI tools. Multiple short comments reflected that many respondents had ‘never used’ or had ‘not tried’ these technologies with some comments combining lack of experience with lack of knowledge; for example: ‘not familiar with chat GPT or other AI, never used them’ (#3, Female, Salaried, N). A few respondents signalled some awareness but lack of experience; for example, “I have never used them. If I was having to write lectures or essays I might?’ (#986, Male, Partner/Principal, N), ‘I’m not too familiar with these but think for information gathering they could be useful…’ (#165, Female, Salaried, N), and ‘I’d be interested in giving it a go.’ (#265, Female, Salaried, N).

#### Theme 2: AI role in clinical practice

##### Optimism

When considering the role of AI tools, optimism was commonly expressed. Many comments were brief, but remarks straddled a broad range of opinions. Most common was the sentiment of guarded optimism suggesting ‘benefits but some concerns’ (#479, Male, Locum, N). For example: ‘correctly used will improve healthcare’ (#59, Male, Partner/Principal, Y) and ‘potential for good, but also big risk of harm’ (#329, Male, Salaried, N). A few GPs were more strident in their enthusiasm; for example: ‘Exciting possibilities,’ (#205, Female, Salaried, N), ‘ChatGPT would be very good in primary care’ (#311, Male, Partner/Principal, Y), ‘Bring it on!’ (#399, Male, Partner/Principal, Y)

##### Documentation and administrative tasks

Some GPs expressed buoyancy about the potential for generative AI in documentation and administrative tasks; for example: ‘Could potentially be immensely useful for documentation, and for collating information from notes,’ (#47, Male, Salaried, Y), and ‘I think it can be excellent in administrative contexts’ (#290, Male, Salaried, N). Some comments described the usefulness of these tools in, ‘letter writing’, for example, ‘improving text used for template letters, for example, letters in support of housing/benefits/education’ (#156, Female, Salaried, Y).

##### Assisting with medical tasks

Some comments suggested generative AI could ‘improve triage’; for instance, ‘They could be a great tool for initial patient screening and care navigation’ (#328, Male, Salaried, Y). The concept of generative AI as a co-pilot for clinical tasks was also identified: comments described these tools as assisting with, or augmenting, medical tasks that doctors would ultimately oversee; for example: ‘I see ChatGPT or other AI as an aide for example spotting patterns of presentations and helping work out optimum monitoring as well as helping with recall’ (#1124, Female, Partner/Principal, Y), ‘list differentials generated by AI, for example, I may not have considered z diagnosis, but I did consider x and y’ (#419, Male, Salaried, N), ‘May be helpful in aiding differential diagnosis, treatment plans and self-care’ (#814, Male, Partner/Principal, N).

#### Theme 3: concerns

##### Deep scepticism

Another prominent category was scepticism about the use of AI in medicine. These comments were brief but emphatic, such as, ‘I have deep concerns’ (#172, Male, Partner/Principal, N), ‘I am very sceptical and nervous about it” (#163, Female, Salaried, N), ‘Absolutely would not use in this context’ (#836, Female, Locum, N), ‘At this stage represents huge risks’ (#713, Male, Partner/Principal, N), ‘It’s crap’ (#536, Male, Partner/Principal, Y).

##### Errors and inaccuracies

Some GPs also offered justifications for this scepticism concerned by alluding to the risks of errors and inaccuracies with generative AI technologies: ‘All the time they make stuff up’ (#252, Female, Salaried, N), ‘It’s not accurate,’ (#321, Female, Locum, N). Only a few GPs offered more developed reflections on these limitations: ‘AI cannot understand context’ (#376, Male, Partner/Principal, N), ‘Looks so authoritative that may be misleading’ (#87, Male, Salaried, Y), ‘AI Language models hallucinate; therefore, cannot be relied on for evidence-based decision making’ (#972, Male, Partner/Principal, Y).

##### Legal concerns

Another concern described by GPs was accountability for mistakes and legal implications for generative AI tools: ‘who will take the blame????’ (#898, Male, Salaried, N), ‘unclear at this stage where liability would lie” (#835, Male, Registrar, N), “Who will take the litigation risk if a physician uses AI tools? If the litigation risk can be reduced or removed, then I think AI would be more welcome’ (#419, Male, Salaried, N). The risks of defensive medicine were also raised: ‘Increased risk of using, that is, a patient will sue me for not doing a million tests that ChatGPT suggested’ (#560, Male, Partner/Principal, Y), ‘I can see LLM output being used in legal action against doctors which will encourage defensive medicine’ (#627, Male, Registrar, N).

#### Theme 4: the future

##### Too early to call

A prominent category was the idea that AI was ‘a fledgling technology’ (#1022, Male, Salaried, N) and at ‘in an infant stage’ (#1066, Male, Partner/Principal, Y). Some GPs believed it was too premature to make a judgment call on the impact on medicine, for example, ‘It is too early to say how AI will change clinical practice’ (#456, Male, Salaried, N), and ‘AI is still quite green. Time will tell what impact will have’ (#746, Male, Locum, Y).

##### Safety research and training

Anticipating a potential future role for AI in medicine, some comments expressed the need for more research into the safety of these technologies. For example: ‘*needs more trials done before used in clinical practice’* (#263, Female, Salaried, N), ‘*Good in theory, needs more improvements to be fully reliable’* (#542, Male, Salaried, N); ‘*exciting developments but my understanding is they need a lot more improvement to reduce hallucinations before could be put into practice’* (#394, Female, Partner/Principal, N)*; ‘More research is needed to assess safety before implementation’* (#62, Prefer not to say, Salaried, N). To future-proof the safe adoption of these tools, some GPs also suggested that more training and education would be needed; for instance: ‘*It will be coming, but I hope we are trained in it by then,’* (#839, Female, Salaried, N), and ‘*More training needed if to be used regularly. How can we use it to our advantage safely”* (#142, Female, Salaried, Y), ‘*Needs training & guidance & time’* (#485, Male, Locum, Y).

##### Doctor–patient interactions

Many comments expressed the opinion that face-to-face interactions between doctors and patients would always be necessary. Some of these comments reflected the view that medical expertise required human interactions; for example, ‘I think the use of AI will remove from general practitioner their ‘gut feeling,’ ‘physical examination,’ ‘history of patient’ and the benefit of the face-to-face assessment’ (#698, Male, Partner/Principal, N), ‘However good these things get, there is still no substitute for that gut-feeing something isn’t right with a patient’ (#722, Female, Salaried, Y). Other comments expressed the view that patients will always desire human interactions: ‘It devalues the significant benefit from someone telling their doctor face to face what they are worried about’ (#396, Female, Salaried, N), ‘a lot of my job is about genuine interpersonal interaction and empathy and I cannot see how AI will achieve this’ (#229, Female, Partner/Principal, N).

## Discussion

Few studies have explored the opinions of doctors about LLM-chatbots on clinical practice. This survey of 1006 practising physicians explored the experiences and opinions of UK GPs about Chat GPT, Bard and Bing AI on their practice. Building on previously published findings from this survey which found 20% (n=205) of UK GPs in the sample reported using generative AI tools in clinical practice^27^; in the present study, we further explored the experiences and opinions of British primary care doctors about these tools. We found that male GPs working in the UK were significantly more likely than female GPs to have used AI, as well as those practising in bigger cities.

Notably, 80% of surveyed GPs at least somewhat agreed that they needed more support/training in understanding these tools. 62% of GPs at least somewhat agreed that more patients would rely on these tools instead of seeking medical attention. While more than a quarter responded that they ‘didn’t know’ what the impact of generative AI would be on care delivery, more than half of surveyed GPs at least somewhat agreed that these tools would increase risks of inequities (55%), and patient harm (54%). In contrast, however, nearly half (47%) believed these tools would increase efficiencies in healthcare.

We found significant differences in respondents’ opinions depending on whether GPs reported using generative AI tools in any aspect of clinical practice. Among those who had used these tools compared with those who had not, GPs were more likely to at least somewhat agree they needed more support and understanding in these tools (87% vs 78%), that patients would rely on these tools before seeking medical attention (70% vs 60%), and these tools would increase efficiencies in care (71% vs 41%). In contrast, those who reported using Chat GPT, Bard or Bing AI to assist with clinical practice were also more likely to at least somewhat agree that these tools could increase inequities in care delivery (57% vs 54%), and patient harm (57% vs 51%), with more who had used AI at least somewhat disagreeing with these statements (27% vs 15% and 27% vs 13%, respectively).

Considering the potential for AI tools to improve tasks in care, GPs at least somewhat agreed that these tools would improve documentation (59%), patient information gathering (56%), the creation of personalised treatment plans (41%), diagnostic accuracy (40%) and prognostic accuracy (38%). Most GPs (65%) at least somewhat disagreed that these tools could improve the delivery of empathy. Again, opinions differed depending on whether GPs reported using generative AI: GPs who had used these tools were more likely to be positive than those who had not, at least somewhat agreeing that these tools would improve documentation (78% vs 54%), patient information gathering (67% vs 53%), the creation of personalised treatment plans (61% vs 35%), diagnostic accuracy (59% vs 35%), prognostic accuracy (51% vs 34%) and the delivery of empathy (29% vs 9%).

Across the whole sample, around one-third (34%) believed that use of these tools would ‘neither decrease nor increase’ the risk of litigation being taken against them. Here again, previous use of AI tools influenced attitudes: 35% of those GPs who reported using generative AI compared with 16% who had not believed these tools would ‘neither decrease nor increase their risk’. Among those who had not used AI, almost half (48%) responded to not knowing the potential impact of AI on litigation, further demonstrating the differences between AI-experienced and inexperienced GPs.

Results from the qualitative section of the survey supported and elaborated on these experiences and opinions. A dominant theme was a lack of deep knowledge, awareness or experience with generative AI tools. Another leading theme was the role of these tools in clinical practice, with many GPs expressing optimism for the potential for benefits in primary care—especially when it comes to documentation and administrative tasks—even if this buoyancy was often tempered with some concerns. Indeed, concerns about risks of clinical errors and inaccuracies, as well as legal anxieties about accountability and blame, comprised another overarching theme. It is notable that despite widespread concerns about data privacy when using commercial generative AI tools,^28^ no respondents explicitly mentioned this in the free-text responses—possibly reflecting limited awareness or understanding of how these tools handle input and potential UK General Data Protection Regulation implications. Reflecting on the future of generative AI in primary care, many GPs expressed uncertainties about the scope for impact, while the need for safety research and physician training was identified as growing needs. Finally, and again supporting the quantitative component of the study, some GPs also expressed the view that doctor–patient interactions would always be necessary to preserve empathy and humanistic aspects of care.

### Comparison with prior work

The results of this study mirror other recent exploratory studies which suggest clinicians may be adopting these tools in greater numbers than anticipated. For example, in one study, more than 1 in 10 surveyed healthcare professionals reported using LLM-model powered chatbots such as ChatGPT, and nearly 50% expressed an intent to use these technologies in the future for tasks such as data entry, medical scheduling or research.^33^ Similarly, in a small survey (n=138) conducted with psychiatrists affiliated to the American Psychiatric Association, 44% of respondents had used ChatGPT 3.5 and 33% had used 4.0 ‘to assist with answering clinical questions’ with 70% of psychiatrists believing that ‘documentation will be/is more efficient’ as a result of these tools.^34^ In that study, 8 in 10 agreed ‘clinicians need more support/training in understanding these tools’ with respondents expressing divergent opinions about the promise and harms associated with these chatbots in healthcare. A survey by the American Medical Association found that US healthcare workers also shared mixed views on AI with the most encouragement around documentation and the most concern around privacy.^30^ While our findings align with the former, privacy concerns were absent in our respondents’ comments. The respondents’ scepticism towards generative AI’s ability to convey empathy is also in contrast to previous research. A previous study of ChatGPT-transformed clinical notes found that generative AI can signal various dimensions of empathy.^10 35^

We also observed some complementary predictions with medical informaticians and other experts working in AI/machine learning (ML) and related fields. Similar to the views of researchers and experts investigating the role of AI healthcare,^36^ many GPs optimistically advanced the view that these tools might function best as ‘co-pilots’ assisting them in a variety of tasks. Like expert informaticians, GPs in our survey expressed the need for more AI training. This education concern echoes the forecasts of a Delphi poll of international health informatics experts who reported consensus that in 10 years (by 2029), advances in AI/ML would prompt workplace changes including incursions into the disintermediation of physician expertise and increased AI/ML training requirements.^24^ While some National Health Service Trusts and professional bodies have issued early position statements,^25 36^ comprehensive national guidelines specific to generative AI in clinical care remain limited.

GPs in our study, especially those who had used generative AI, believed patients would increasingly use these tools. Notably, experts in the aforementioned international Delphi poll predicted that by 2029, AI and ML will significantly enhance diagnostic accuracy, particularly benefiting individuals with limited access to specialists, minority groups and those with rare diseases; and improved access to expert doctor knowledge.^24^ Similarly, in another study, 8 in 10 Americans surveyed believed tools such as ChatGPT had the potential to improve the quality of healthcare, reduce costs and increase the accessibility of care.^33^

### Strengths and limitations

The main strength of this survey is that it is the largest conducted anywhere exploring clinicians’ opinions about the impact of generative AI on medicine. The use of a web-based survey may have facilitated honest feedback about UK GPs’ views on generative AI, as reflected in the candour of our participants’ responses.

The study has several limitations. The use of a convenience sample means that it is not possible to infer that our sample was representative of the opinions of GPs in the UK. Although we strove to stratify the sample according to geographic location, our respondents differed from those registered with the GMC. While our sample closely resembled the geographical representation of UK GPs, our sample was younger and included more male GPs. Our respondents were restricted to those GPs who used Doctors.net.uk, including during the administration of the survey, and response biases may have influenced the findings. For example, it is possible that GPs with stronger (negative or positive) views about generative AI may have been more inclined to participate. Selection bias can also be a contributory factor explaining the majority of male GPs observed in the sample, in contrast to the majority of females in the underlying GMP population. In addition, comments in the qualitative component of the survey were often brief owing to the constraints of the web-based survey design. As noted in the Methods, we specifically restricted the survey to opinions about commercially available LLM-powered chatbots, but respondents may have had different opinions about medical grade models such as Google’s PALMMed2 and this should be explored further too.

In the current study, we chose to evaluate three specific models: Chat GPT/Bard/Bing AI. We believe it is a reasonable choice, owing to the public renown and availability of these models. Naming the models was also deemed necessary, not to confuse other types of AI with generative AI. However, it is possible that more advanced AI-users, aware of the privacy concerns, instead use locally hosted open-weights models such as iterations of LLama3 or Mistral AI. This limitation may bias the results towards a more positive view of AI use, as users of open-source models—who may be more privacy-conscious or technically critical—could be underrepresented in the sample.

More robust, nationally representative surveys are required to obtain a clearer picture of doctors’ views and experiences with generative AI. Particularly, on the acceptability of applying generative AI in patient communication as a method to cue empathy in clinical documentation. In addition, future research could usefully engage the expert opinions of health informaticists, medical AI researchers and ethicists in relation to the use of medical-grade generative AI in clinical practice. Finally, more extensive, stand-alone survey research is required to understand the perspectives of patients using generative AI, including their opinions about doctors using these tools.

## Conclusions

This study provides exploratory insights into the views of 1006 GPs in the UK regarding their experiences and opinions about generative AI in patient care and in their practice. There was an over-riding shared consensus among GPs that they needed more support/training in understanding generative AI tools. Notably, however, GPs who used these tools were significantly more optimistic about the scope for generative AI in improving clinical tasks compared with those who did not report using them. GPs who adopted these tools were more likely to agree that generative AI could improve documentation, patient information gathering, the creation of personalised treatment plans, diagnostic accuracy, prognostic accuracy and empathic care. These GPs were also more likely to believe generative AI would improve efficiencies in care.

Conspicuously, however, GPs held disparate opinions about the litigation risks of using these tools, including when it came to accountability if things went wrong. Many GPs expressed lack of knowledge and experience with these tools. Lack of awareness was most notable in the absence of discussion about the patient privacy concerns with the adoption of these tools.

We conclude that many doctors may already be using generative AI at scale and finding use for these tools in clinical practice. We caution that medical organisations must urgently invest more sustained efforts in educating and guiding physicians on the safe adoption and limitations of generative AI in patient care. We suggest that relevant federal and national medical organisations also make it clear to physicians what types of generative AI can be used and how.

## Data Availability

Data are available on reasonable request.
