# Supplementary material for: Generative artificial intelligence in medicine: a mixed-methods survey of UK general practitioners
Source: BMJ Digit Health Ai. 2025 Jul 23;1(1):e000051. doi: 10.1136/bmjdhai-2025-000051 (PMC13528321; doi:10.1136/bmjdhai-2025-000051)
Supplement: Supplementary file 2 [file bmjdhai-1-1-bmjdhai-2025-000051-s002.pdf]

Appendix 2. Survey

Part A

S1 - Role

Which of the following best describes your role?

❍ GP Partner / Principal (1)
❍ Salaried GP (2)
❍ Locum GP (3)
❍ GP Registrar (4)
❍ Secondary Care doctor (5)
❍ Other (6)

S2 - Country/region

Where are you currently practising?

❍ London (1)
❍ South West (2)
❍ South East (3)
❍ West Midlands (4)
❍ East Midlands (5)
❍ East of England (6)
❍ Yorkshire and Humber (7)
❍ North East (8)
❍ North West (9)
❍ Scotland (10)
❍ Wales (11)
❍ Northern Ireland (12)
❍ Not currently working (13)
❍ Working outside the UK (14)

D1 - Gender

Are you...

❍ Male (1)
❍ Female (2)
❍ Other (3)
❍ Prefer not to say (4)

D2 - Age

Are you...

❍ 35 or under (1)
❍ 36 to 45 (2)
❍ 46 to 55 (3)
❍ 56 or over (4)

Kharko A, *et al. BMJ Digit Health* 2025; 1:e000051. doi: 10.1136/bmjdhai-2025-000051

D4 - Practice Size

How many patients do you have on your practice list?

❍ Up to 5,000 patients (1)
❍ 5,001-7,500 patients (2)
❍ 7,501-10,000 patients (3)
❍ 10,001-12,500 patients (4)
❍ 12,501 patients or more (5)

D5 - Year of qualification

When did you qualify as a doctor?

❍ 1960 (1960)
❍ 1961 (1961)
❍ 1962 (1962)
❍ 1963 (1963)
❍ 1964 (1964)
❍ 1965 (1965)
❍ 1966 (1966)
❍ 1967 (1967)
❍ 1968 (1968)
❍ 1969 (1969)
❍ 1970 (1970)
❍ 1971 (1971)
❍ 1972 (1972)
❍ 1973 (1973)
❍ 1974 (1974)
❍ 1975 (1975)
❍ 1976 (1976)
❍ 1977 (1977)
❍ 1978 (1978)
❍ 1979 (1979)
❍ 1980 (1980)
❍ 1981 (1981)
❍ 1982 (1982)
❍ 1983 (1983)
❍ 1984 (1984)
❍ 1985 (1985)
❍ 1986 (1986)
❍ 1987 (1987)
❍ 1988 (1988)
❍ 1989 (1989)
❍ 1990 (1990)
❍ 1991 (1991)
❍ 1992 (1992)
❍ 1993 (1993)
❍ 1994 (1994)
❍ 1995 (1995)

Kharko A, *et al. BMJ Digit Health* 2025; 1:e000051. doi: 10.1136/bmjdhai-2025-000051

❍ 1996 (1996)
❍ 1997 (1997)
❍ 1998 (1998)
❍ 1999 (1999)
❍ 2000 (2000)
❍ 2001 (2001)
❍ 2002 (2002)
❍ 2003 (2003)
❍ 2004 (2004)
❍ 2005 (2005)
❍ 2006 (2006)
❍ 2007 (2007)
❍ 2008 (2008)
❍ 2009 (2009)
❍ 2010 (2010)
❍ 2011 (2011)
❍ 2012 (2012)
❍ 2013 (2013)
❍ 2014 (2014)
❍ 2015 (2015)
❍ 2016 (2016)
❍ 2017 (2017)
❍ 2018 (2018)
❍ 2019 (2019)
❍ 2020 (2020)
❍ 2021 (2021)
❍ 2022 (2022)

D3 - Practice

Which of the following best describes the area where your practice is based?

❍ Major conurbation (e.g. London, Glasgow) (1)
❍ Large town/city (e.g. Nottingham, Cardiff) (2)
❍ Medium town/city (e.g. Worcester, Dundee) (3)
❍ Small town/city (e.g. Thetford, Omagh) (4)
❍ Village/hamlet (5)
❍ Other (6)

D3b

Please specify “other” practice location

____

D5a - GPwER

Are you a GP with an extended role (GPwER) in any of the following areas ?

❑ Acute medicine (1)
❑ Cardiology (2)
❑ Dermatology (3)

Kharko A, *et al. BMJ Digit Health* 2025; 1:e000051. doi: 10.1136/bmjdhai-2025-000051

❑ Diabetes (4)
❑ Mental Health (5)
❑ Musculoskeletal healthcare / sports injuries (6)
❑ Neurology (7)
❑ Reproductive health (8)
❑ Respiratory medicine (9)
❑ Women's health (10)
❑ Other (98)
❍ None of these (99)

Part B

The following questions ask for your experiences with using Open AI's Chat GPT (or Google's Bard or Microsoft's Bing AI if you have used those instead):

Q1

Have you ever used any of the following to assist you in any aspect of clinical practice? Please select all that apply:

❑ Chat GPT (1)
❑ Google's Bard (2)
❑ Microsoft's Bing AI (3)
❑ Other (please specify) (4)________________
❍ None (5)

Q1a

What are you using the tool to assist with? Please select all that apply:

❑ Suggesting a differential diagnosis (1)
❑ Suggesting treatment options (2)
❑ Generating documentation after patient appointments (3)
❑ Patient summarization / timelines from prior documentation (4)
❑ Other (please specify) (5)________________

Part C

Q2

Please think about how the following tasks could be affected by Chat GPT/Bard/Bing AI. Indicate how strongly you agree or disagree with the statements below.

Kharko A, *et al. BMJ Digit Health* 2025; 1:e000051. doi: 10.1136/bmjdhai-2025-000051

"These tools will improve…

	Strongly disagree (1)	Disagree (2)	Somewhat disagree (3)	Somewhat agree (4)	Agree (5)	Strongly agree (6)	Don't know (7)
patient information gathering (1)	□	□	□	□	□	□	□
diagnostic accuracy (2)	□	□	□	□	□	□	□
the creation of personalized treatment plans (3)	□	□	□	□	□	□	□
prognostic accuracy (4)	□	□	□	□	□	□	□
the delivery of empathy (5)	□	□	□	□	□	□	□
documentation (6)	□	□	□	□	□	□	□

Q3

Please think about how healthcare could be affected by Chat GPT/Bard/Bing AI. Indicate how strongly you agree or disagree with the statements below which refer to the use of these tools among clinicians and patients.

"Use of these tools will…

	Strongly disagree (1)	Disagree (2)	Somewhat disagree (3)	Somewhat agree (4)	Agree (5)	Strongly agree (6)	Don't know (7)
increase the risk of patient harm (1)	□	□	□	□	□	□	□
increase risks of inequities in care delivery (2)	□	□	□	□	□	□	□
mean more patients will rely on them instead of seeking medical attention (3)	□	□	□	□	□	□	□

Kharko A, *et al. BMJ Digit Health* 2025; 1:e000051. doi: 10.1136/bmjdhai-2025-000051

	Strongly disagree (1)	Disagree (2)	Somewhat disagree (3)	Somewhat agree (4)	Agree (5)	Strongly agree (6)	Don't know (7)
mean clinicians need more support/training in understanding them (4)	☐	☐	☐	☐	☐	☐	☐
Increase efficiencies in healthcare (5)	☐	☐	☐	☐	☐	☐	☐

Q4

Please think about how your practice could be affected by Chat GPT/Bard/Bing AI.

	Decrease my risk of having legal action taken against me (1)	Increase my risk of having legal action taken against me (2)	Neither decrease nor increase my risk (3)	Don't know (4)
In your opinion, these tools will: (1)	☐	☐	☐	☐

Q5

Please add any additional comments you might have about the use of Chat GPT/Bard/Bing AI in primary care.

Kharko A, *et al. BMJ Digit Health* 2025; 1:e000051. doi: 10.1136/bmjdhai-2025-000051

BAS5x99

❑ No additional comments (99)

D3

Please add any additional comments you might have about the use of the Chat GPT/Bard/Bing AI in medicine.

D3ax99

❑ No additional comments (99)

Kharko A, *et al. BMJ Digit Health* 2025; 1:e000051. doi: 10.1136/bmjdhai-2025-000051
