# Supplementary material for: Generative artificial intelligence in medicine: a mixed-methods survey of UK general practitioners
Source: BMJ Digit Health Ai. 2025 Jul 23;1(1):e000051. doi: 10.1136/bmjdhai-2025-000051 (PMC13528321; doi:10.1136/bmjdhai-2025-000051)
Supplement: Supplementary file 3 [file bmjdhai-1-1-bmjdhai-2025-000051-s003.pdf]

Appendix 3. Additional analyses

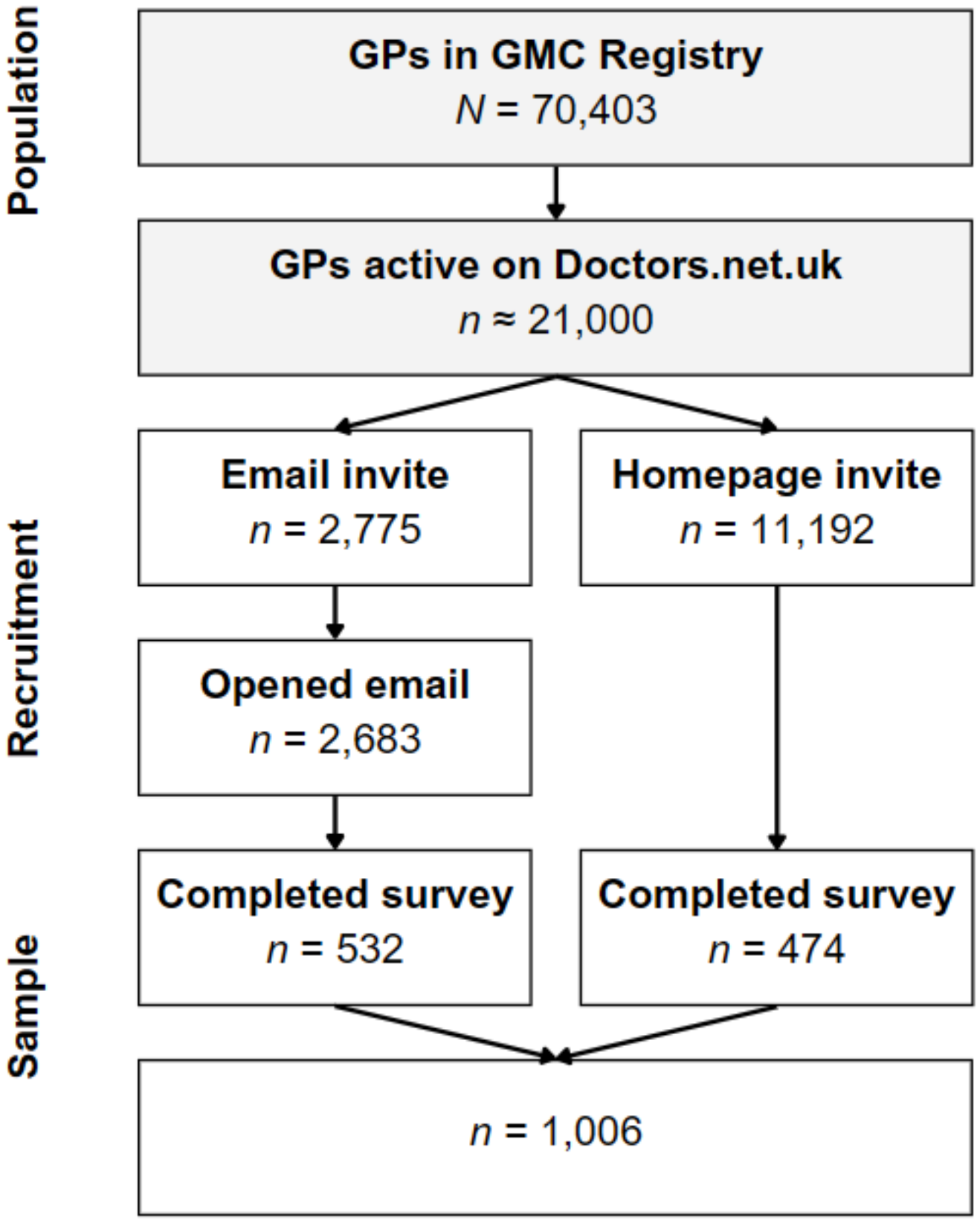

Figure 1. Study flow.

Kharko A, *et al. BMJ Digit Health* 2025; 1:e000051. doi: 10.1136/bmjdhai-2025-000051

Comparison of the survey sample to GPs in the GMC Registry

Table 1. Gender

	**Sample** (n = 1,006)	**GMC Registry** (n = 70,403)
**Woman**	467 (46.42%)	40,725 (58%)
**Man**	531 (52.78%)	29,678 (42%)

Table 2. Age range

	**Sample** (n = 1,005)	**GMC Registry** (n = 70,403)
**35 years or younger**	76 (7.55%)	7,638 (11%)
**35 – 44 years**	386 (38.37%)	26,275 (37%)
**45 – 54 years**	348 (34.59%)	20,804 (30%)
**55 years or older**	196 (19.48%)	15,686 (22%)

Note: Age ranges in the survey were *'35 or younger'*, *'36 – 45'*, *'56 – 55'*, and *'56 or older'*.

Table 3. Regional distribution of sample GPs compared to those in GMC Registry.

	**Sample** (n = 1,006)	**GMC Registry** (n = 70,403)
**England**		
North West	121 (12.03%)	7,618 (11%)
North East and Yorkshire	131 (13.02%)	8,460 (12%)
Midlands	162 (16.1%)	10,386 (15%)
East of England	84 (8.35%)	5,982 (8%)
London	113 (11.23%)	10,351 (15%)
South East	130 (12.92%)	9,319 (13%)
South West	99 (9.84%)	6,685 (10%)
**Northern Ireland**	34 (3.38%)	2,025 (3%)
**Scotland**	84 (8.35%)	6,633 (9%)
**Wales**	48 (4.77%)	2,944 (4%)

Kharko A, *et al. BMJ Digit Health* 2025; 1:e000051. doi: 10.1136/bmjdhai-2025-000051

GPs' characteristics associated with use of generative AI

$$[\text{Used AI} = b_0 + b_1 \times \text{Gender} + b_2 \times \text{Practice Location} + \epsilon]$$

$$\text{Used\_AI} = \beta_0 + \beta_1 \times \text{Location}_{\text{Major conurbation}} + \beta_2 \times \text{Location}_{\text{Large town/city}} + \beta_3 \text{ Location}_{\text{Small town/city}} + \beta_4 \times \text{Location}_{\text{Village/hamlet}} + \beta_5 \times \text{Location}_{\text{Other}} + \beta_6 \times \text{Gender}_{\text{Male}} + \epsilon$$

Equation 1. Best model predicting whether a GP used AI or not.

Table 4. Model statistics for GP characteristics affecting use of AI in clinical practice

Variable	**Coefficient**	**Standard Error**	***t-value***	***p-value***
(Intercept)	.207	.03	7.03	< .001 ***
**Male**	.089	.03	3.54	< .001 ***
**Major conurbation**	- .015	.04	- .40	= .694
**Large town/city**	- .008	.04	- .20	= .845
**Small town/city**	- .092	.04	- 2.6	= .009 **
**Village/hamlet**	- .158	.05	- 3.5	< .001 ***
**Other**	- .274	.14	- 2.0	= .055 .

Note: *** indicates p < .001; ** indicates p < .01; * indicates p < .05; . indicates p < .1

Kharko A, *et al. BMJ Digit Health* 2025; 1:e000051. doi: 10.1136/bmjdhai-2025-000051

GPs' opinions about the impact of Generative AI

Table 5. Results from Chi-square of Independence comparing GPs' opinions about impact of generative AI based on prior use.

Item	χ^2	*df*	Cramer's V	*p*-value
**AI tools will improve …**				
documentation.	62.1	6	.2	< .001
patient information gathering.	43.2	6	.2	< .001
diagnostic accuracy.	56.3	6	.2	< .001
the creation of personalised treatment plans.	58.5	6	.2	< .001
prognostic accuracy.	46.5	6	.2	< .001
the delivery of empathy.	65.8	6	.3	< .001
**Use of AI tools will …**				
mean clinicians need more support/training in understanding them.	23.0	6	.2	< .001
increase efficiencies in healthcare.	68.9	6	.3	< .001
mean more patients will rely on them instead of seeking medical attention.	32.5	6	.2	< .001
increase risks of inequities in care delivery.	34.1	6	.2	< .001
increase the risk of patient harm.	47.8	6	.2	< .001

Note: Significance level for p-value was pre-set to $p < .05$.

Table 6. Results from Chi-square of Independence comparing GPs' opinions about litigation risks based on prior use of generative AI.

Item	χ^2	*df*	Cramer's V	*p*-value
**Litigation risk**	66.7	3	.3	< .001

Note: Significance level for p-value was pre-set to $p < .05$.

Kharko A, *et al. BMJ Digit Health* 2025; 1:e000051. doi: 10.1136/bmjdhai-2025-000051

GPs' characteristics associated with opinions about Generative AI

Table 7. Comparison of median opinion ratings between women and men.

Item	**Women** (*n* = 467)	**Men** (*n* = 531)	Mann-Whitney U Test Results
**AI tools will improve …**			
documentation.	4	4	$U = 68734$, $p = .626$
patient information gathering.	4	4	$U = 62926$, $p = .01$
diagnostic accuracy.	4	4	$U = 55726.5$, $p = .184$
the creation of personalised treatment plans.	3	4	$U = 63385$, $p = .137$
prognostic accuracy.	4	4	$U = 53034.5$, $p = .109$
the delivery of empathy.	2	2	$U = 74932.5$, $p = .115$
**Use of AI tools will …**			
mean clinicians need more support/training in understanding them.	5	5	$U = 96022$, $p = .08$
increase efficiencies in healthcare.	4	4	$U = 62126.5$, $p = .53$
mean more patients will rely on them instead of seeking medical attention.	4	4	$U = 76440.5$, $p = .839$
increase risks of inequities in care delivery.	4	4	$U = 73174$, $p < .001$
increase the risk of patient harm.	4	4	$U = 66947$, $p = .015$

Note: *'Don't know'* responses were excluded from calculations. The numerical ratings had the following labels: 1 – *'Strongly disagree'*, 2 – *'Disagree'*, 3 – *'Somewhat disagree'*, 4 – *'Somewhat agree'*, 5 – *'Agree'*, 6 – *'Strongly agree'*.

Table 8. Comparison of median opinion ratings between younger (≤ 45 years) and older (≥ 46 years) GPs.

Item	**Women** (*n* = 467)	**Men** (*n* = 531)	Mann-Whitney U Test Results
**AI tools will improve …**			
documentation.	4	4	$U = 68431.5$, $p = .335$
patient information gathering.	4	4	**$U = 78704$, $p = .008$**
diagnostic accuracy.	4	4	$U = 63986.5$, $p = .117$
the creation of personalised treatment plans.	4	4	**$U = 76768.5$, $p = .003$**
prognostic accuracy.	4	4	$U = 58969.5$, $p = .718$
the delivery of empathy.	2	2	**$U = 86711$, $p = .039$**
**Use of AI tools will …**			
mean clinicians need more support/training in understanding them.	5	5	$U = 93310.5$, $p = .344$
increase efficiencies in healthcare.	4	4	$U = 60690$, $p = .12$
mean more patients will rely on them instead of seeking medical attention.	4	4	$U = 73443$, $p = .403$
increase risks of inequities in care delivery.	4	4	$U = 62163.5$, $p = .502$
increase the risk of patient harm.	4	4	$U = 57844.5$, $p = 280$

Note: *'Don't know'* responses were excluded from calculations. The numerical ratings had the following labels: 1 – *'Strongly disagree'*, 2 – *'Disagree'*, 3 – *'Somewhat disagree'*, 4 – *'Somewhat agree'*, 5 – *'Agree'*, 6 – *'Strongly agree'*.

Kharko A, *et al. BMJ Digit Health* 2025; 1:e000051. doi: 10.1136/bmjdhai-2025-000051

Comparison of respondents who did and did not leave comments

Table 9. Comparison of respondents who did and did not leave comments across several respondent characteristics.

Variable	**Comments**	**No comments**	Chi-square Test Results
**Gender**			χ^2 (2, 1006) = 4.32, *p* = .12
Woman	148 (47.74%)	319 (45.83%)	
Man	157 (50.65%)	374 (53.74%)	
Prefer not to say	5 (1.61%)	3 (.43%)	
**Age**			χ^2 (3, 1006) = 7.91, *p* = .052
35 yrs or younger	26 (8.39%)	50 (7.18%)	
36 – 45 years	100 (32.26%)	286 (41.09%)	
46 – 55 years	113 (36.45%)	235 (33.76%)	
56 yrs or older	71 (22.9%)	125 (17.96%)	
**Experience with AI**			χ^2 (1, 1006) = 7.20, *p* < .001
Used AI	79 (25.48%)	126 (18.1%)	
Did not use AI	231 (74.52%)	570 (81.9%)	
**Clinical practice size**			χ^2 (4, 1006) = 10.67, *p* = .03
Up to 5,000 patients	42 (13.55%)	75 (10.78%)	
5,001 – 7,500 patients	34 (10.97%)	115 (16.52%)	
7,501 – 10,000 patients	78 (25.16%)	135 (19.4%)	
10,001 – 12,500 patients	49 (15.81%)	134 (19.25%)	
12,501 patients or more	107 (34.52%)	237 (34.05%)	

Kharko A, *et al. BMJ Digit Health* 2025; 1:e000051. doi: 10.1136/bmjdhai-2025-000051
